# Distinct Neutrophil Populations in the Spleen During PICS

**DOI:** 10.3389/fimmu.2020.00804

**Published:** 2020-05-15

**Authors:** Satarupa Sengupta, Charles C. Caldwell, Vanessa Nomellini

**Affiliations:** ^1^Division of Research, Department of Surgery, University of Cincinnati College of Medicine, Cincinnati, OH, United States; ^2^Division of Research, Shriners Hospital for Children, Cincinnati, OH, United States; ^3^Section of General Surgery, Department of Surgery, University of Cincinnati College of Medicine, Cincinnati, OH, United States

**Keywords:** sepsis, PICS, neutrophils, CD54, immunosuppression

## Abstract

While mortality after acute sepsis has decreased, the long-term recovery for survivors is still poor, particularly those developing persistent inflammation, immunosuppression, and catabolism syndrome (PICS). While previously thought that activated neutrophils responding to the acute phase of sepsis migrate to the spleen to undergo cell death and contribute to immunosuppression, our data show a significant accumulation of distinct, yet functional, neutrophil populations in the spleen in a murine model of PICS. The exact role and function of neutrophils in this response is still unclear. The objective of our study was to better define the immune function of splenic neutrophils to determine if this could give insight into the pathogenesis of PICS. Using a murine model of cecal ligation and puncture (CLP), which demonstrates all characteristics of PICS by 8 days, spleens were harvested, and neutrophils were identified by Ly6G and CD11b expression via flow cytometry. Nearly all splenic neutrophils expressed CD54, but there were distinct CD54^hi^ and CD54^lo^ cells, with the majority being CD54^lo^ cells during PICS. The CD54^hi^ population showed traditional, proinflammatory properties, but a relatively decreased chemotactic response, while CD54^lo^ cells had significantly higher chemotaxis, yet significantly decreased proinflammatory functions. Using 5-ethynyl-2′-deoxyuridine (EdU) incorporation, we found that the CD54^hi^ population on day 2 after CLP may be participating in emergency myelopoiesis. However, the vast majority of the CD54^lo^ population were paused in the G_1_ phase at this time point and not proliferating. By day 8 after CLP, most of the CD54^hi^ cells in the spleen were no longer proliferating, while the CD54^lo^ cells were, indicating that CD54^lo^ dominate in extramedullary myelopoiesis at later time points. Almost none of the neutrophils produced arginase or inducible nitric oxide synthase (iNOS), indicating that these are not suppressor cells. Overall, our data demonstrate that neutrophil accumulation in the spleen during PICS is related to extramedullary myelopoiesis, leading to the production of immature neutrophils. While not suppressor cells, the majority have greater chemotactic function but less inflammatory responsiveness, which may contribute to the immunosuppression seen in PICS. Attention to these distinct neutrophil populations after septic or other systemic inflammatory responses is therefore critical to understanding the mechanisms of PICS.

## Introduction

Recent advancements in the initial diagnosis and management of sepsis have resulted in improved overall survival. However, the long-term recovery among sepsis survivors is still poor, often leading to a state of chronic critical illness (1). This condition is frequently associated with a compromised immune system, also called persistent inflammation, immunosuppression, and catabolism syndrome (PICS) (2). As a result, these patients suffer from multiple complications, poor wound healing, increased disability, and susceptibility to secondary infections leading to prolonged hospitalizations (3). Despite extensive care and intervention, ~50% of chronic critically ill patients die within 6 months of ICU discharge, and for those that are able to survive to 1 year after discharge, at least 20% show significant physical and cognitive disabilities, with almost 10% never returning home (3, 4). Failure of therapeutic interventions for sepsis-associated chronic critical illness is largely due to the insufficient information available about the immune dysfunction that occurs after sepsis.

Neutrophils are the key responders to infection in that activated neutrophils are recruited to the site of bacterial invasion to fulfill their antimicrobial function (5). Historically, it was thought that, following bacterial clearance, neutrophils mostly migrate to the spleen to undergo cell death, while the bone marrow undergoes emergency myelopoiesis to regenerate the neutrophil population (6). However, our data show a significant accumulation of distinct, yet functional, neutrophil populations in the spleen in a murine model of PICS, suggesting a possible role for these cells in secondary infections and/or the overall systemic response to sepsis.

Neutrophil rolling and migration involves the transmembrane glycoprotein and adhesion molecule, L-selectin (CD62L) in conjunction with β_2_-integrin activation and adhesion to counter-receptors such as intracellular adhesion molecules (ICAM-1) (CD54) (7, 8). The ectodomain shedding of CD62L from neutrophil plasma membrane denotes neutrophil activation or partial activation (priming), concordant with upregulation of CD11b, a component of the macrophage-1 antigen (Mac-1) (CD11b/CD18) β_2_-integrin subfamily (9). Appearance of the surface marker, CD54, on activated neutrophils correlates with reverse transendothelial migration, and its expression is known to be increased by inflammatory stimuli (10, 11). Neutrophils showing antitumorigenic phenotypes show increased CD54 expression (12), while CD54 expressing neutrophils are also associated with chronic systemic inflammation (13). However, the functional properties of these neutrophil subpopulations remain elusive (14). In our study utilizing a murine PICS model, we found that the myeloid-derived splenic neutrophils (Ly6G^+^CD11b^+^) distinctly comprised two populations based on the surface CD54 expression. We therefore decided to pursue this further to characterize the CD54 subpopulations (CD54 high and low) to help understand the immunosuppression in PICS. While it is known that the spleen can act as a site of extramedullary myelopoiesis, the exact role and functional properties of these splenic neutrophils is still not clear. Therefore, the objective of our study was to better characterize and define the immune function of these neutrophil subpopulations to gain insight into and better understand the pathogenesis of PICS.

## Materials and Methods

### Cecal Ligation and Puncture Model

Cecal ligation and puncture was performed on 6 to 8-week-old male CD-1 mice from the Charles River Laboratories (Wilmington, MA, USA) as described previously (15). The animal protocol was approved under the Institutional Animal Care and Use Committee of the University of Cincinnati (Protocol No. 10-05-10-01). Briefly, the animals were provided with regular pellet diet and water *ab libitum* and were allowed to acclimatize for 1–2 weeks before experiments in standard environmental conditions. Acute polymicrobial sepsis was induced in the mice by 33% cecal ligation with a single, full-thickness 25-gauge needle puncture under 2.5% isoflurane followed by 3 and 24 h post-surgery primaxin administration. Time of surgery was kept consistent between experiments. The mortality rate remained 25–33% for 3 days after this cecal ligation and puncture (CLP) injury in mice, comparable to the 10–40% in human sepsis cases as defined previously (16, 17).

### Persistent Inflammation, Immunosuppression, and Catabolism Syndrome Model

Mice that survived 8 days after CLP injury and displayed the syndromes including weight loss, lymphocyte depletion, increase in circulating myeloid cells, etc. were used in experiments as PICS mice as described previously (16). Untouched mice were used as control, as they have near-identical levels of systemic inflammation and coagulation parameters 8 days after sham surgery, which includes anesthetic administration and laparotomy without intervention.

### Spleen Harvest and Cell Counts

Spleens were removed from untouched and PICS mice, weighed, and then homogenized in Roswell Park Memorial Institute (RPMI) medium followed by passing through a 70-μm cell strainer (Corning, MA, USA) to obtain a uniform single cell suspension. The total number of white blood cells (WBCs) was enumerated with a cell counter (Beckman Coulter, CA, USA). One to two million cells were used for further characterization of the splenic neutrophil compartment by flow cytometry.

### Flow Cytometry

Flow cytometry was performed on the Attune NxT Flow Cytometer (Life Technologies, CA, USA). Cells were first gated for doublet exclusion [forward scatter height (FSC-H) vs. forward scatter area (FSC-A)] followed by side scatter height (SSC-H) vs. FSC-H gating. Cell viability was checked by negative gating of cells stained with “Live/Dead Fixable Aqua Dead Cell Staining Kit” (Life Technologies, CA, USA). Neutrophils were analyzed by detecting the surface antigens with the following antibodies: Ly6G (clone 1A-8, BD Biosciences, CA, USA), CD11b (clone M1/70, Biolegend, CA, USA), CD54 (clone 3E2), and CD62L (clone MEL-14) from BD Pharmingen, CA, USA; or total antigens (surface and intracellular) by antibodies: CXCR4 (clone L276F12) and CXCR2 (clone SA045E1) from Biolegend, CA, USA; or by intracellular labeling with antibodies: Arg-1 (clone A1exF5) and inducible nitric oxide synthase (iNOS) (clone CXNFT) from Invitrogen, MA, USA. Cells were fixed with 1% paraformaldehyde and permeabilized with Saponin buffer [0.1% Saponin (*w*/*v*), 0.1% bovine serum albumin (BSA), 0.01 M HEPES, and 0.1% sodium azide in phosphate-buffered saline (PBS)] prior to the intracellular labeling as described previously (18).

### Functional Assays

#### DHR Assay

Dihydrorhodamine (DHR) 123 assay was performed to measure the formation of oxidized rhodamine 123 from the non-fluorescent DHR 123, thus to assess reactive oxygen species (ROS) production. Harvested spleen cells were resuspended in Hank's balanced salt solution (HBSS) (Ca^++^Mg^++^) and were incubated with DHR (Sigma, MO, USA) (final 1×) at 37°C for 10 min. The reaction was stopped in ice, and the cells were washed twice with ice-cold fluorescence-activated cell sorting (FACS) buffer (1×). Finally, the cells were labeled with fluorescence-conjugated antibodies against the surface markers of interest (Ly6G, CD11b, CD54), and flow cytometry analysis was performed to detect the green fluorescence of rhodamine 123 as a ROS indicator as described previously (16).

#### pHrodo Assay

pHrodo Green *Escherichia coli* BioParticles Conjugate for Phagocytosis (Invitrogen, MA, USA) were reconstituted in a glass tube and then sonicated in a water bath sonicator for 5 min. Opsonizing reagent was added (1:40) to the *E. coli* BioParticles and was incubated at 37°C for 1 h. The particles were washed twice with PBS, and 100 μl PBS resuspension was added to 1 million splenocytes followed by incubation in 37°C 5% CO_2_ incubator for another hour. The reaction was stopped in ice, and the cells were fixed with 1% paraformaldehyde (PFA). After washing, the cells were labeled with antibodies against the surface markers of interest as described above, and finally, the phagocytosing cells were detected by measuring the green fluorescence uptake of the *E. coli* BioParticles as described previously (19).

#### NETosis Assay

NETosis assay was performed as described previously (20). Briefly, cells were resuspended in RPMI and were stimulated with 100 nM phorbol-12-myristate-13-acetate (PMA) (Sigma, MO, USA) for 3 h at 37°C 5%CO_2_ incubator. Cells were then washed and fixed with 1% PFA followed by further wash, blocking, and staining with primary H3 antibody (1:300, Abcam, MA, USA) for 30 min at room temperature. Then, the cells were incubated with the antibody cocktail of Alexa Fluor700-conjugated secondary antibody (1:300, Invitrogen, MA, USA) and fluorescein isothiocyanate (FITC)-conjugated antimyeloperoxidase (1:50, Abcam, MA, USA), along with the surface markers of interest as described above at room temperature for 30 min in the dark. Finally, the cells were washed and resuspended in FACS buffer for flow cytometry analysis as mentioned (20).

### Chemotaxis Assay

After harvesting and cell counting, 2 million spleen WBCs were seeded on a Transwell insert (Thermo Fisher Scientific, MA, USA) of 3 μm pore size. One hundred nanograms of KC, as a main neutrophil chemoattractant, was added to each of the bottom wells, and the cells were incubated at 37°C CO_2_ incubator for 3 h. Non-migrated cells from the upper Transwell insert and migrated cells from the bottom well were recovered to analyze further by flow cytometry. The percent of cells migrating to the bottom was then calculated as described previously (21).

### Cell Cycle and Proliferation Assays

For cell cycle analysis, the splenocytes were labeled with fluorescence-conjugated antibody against Ki-67 (clone 16A8, Biolegend, CA, USA) and propidium iodide (PI) solution (25 μg/ml) followed by flow cytometry analysis as described previously (22). For the 5-ethynyl-2′-deoxyuridine (EdU) assay, mice were injected with EdU on day 7 after CLP, and the splenocytes were harvested on post-CLP day 8. EdU incorporation into newly synthesized DNA was measured by analyzing the cells using iClick EdU Andy Fluor 488 Flow Cytometry Assay Kit (ABP Biosciences, MD, USA).

### Statistical Analyses

All analyses were performed using the software GraphPad Prism 8 (La Jolla, CA, USA). Student's *t*-test was performed to compare groups, and one-, two-, or a three-way ANOVA was performed for multiple comparisons as applicable. Data were reported as means ± SEM values. Any *p* ≤ 0.05 was considered statistically significant.

## Results

### The Spleen Harbors Both CD54-High and CD54-Low Expressing Neutrophils in PICS Mice

**Figure 1:**
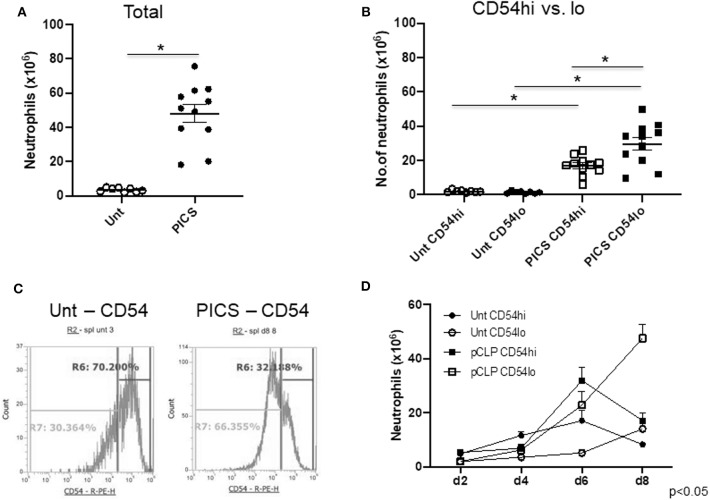
The spleen harbors both CD54-high and CD54-low expressing neutrophils in persistent inflammation, immunosuppression, and catabolism syndrome (PICS) mice. Spleens were harvested from untouched (Unt) mice or mice post-CLP (pCLP) and single cell suspensions were colabeled with Ly6G, CD11b, and CD54 antibodies followed by flow cytometry analysis at different time points. Scatter plots depicting **(A)**, the total number of neutrophils (Ly6G+ CD11b+) in the spleen of Unt and pCLP day 8 (PICS) mice and **(B)** the number of neutrophils expressing high CD54 (CD54^hi^) and low CD54 (CD54^lo^) in Unt and PICS spleens. Black bars indicate the mean ± SEM values. *p < 0.05 was considered significant. **(C)** Histograms demonstrating CD54 expression pattern in splenic neutrophils with high (R6 gate) or low (R7 gate) expression in representative mice (Unt, left; PICS, right). Experiments were repeated at least three times. **(D)** Line graph showing the number of neutrophils with CD54^hi^ or lo expression in the spleen of Unt and CLP mice at day 2 (d2), day 4 (d4), day 6 (d6), and day 8 (d8). A three-way ANOVA analysis of the Unt vs. pCLP, CD54^hi^ vs. CD54lo, and time (days) rendered the data significant (*p* < 0.05).

Single-cell suspensions of spleens from untouched (Unt) mice and mice post-CLP (cecal ligation and puncture) from different days were labeled with neutrophil and myeloid markers (Ly6G and CD11b, respectively) to detect mature neutrophils. Spleen-to-body mass ratio was also quantified to confirm the gradual increase in spleen mass in CLP mice compared to the healthy ones (Figure S1). The total number of neutrophils was significantly increased in PICS mice (post-CLP day 8) compared to the Unt mice (Figure 1A). Furthermore, the neutrophils in PICS spleens were analyzed based on CD54 surface marker expression. While most of the splenic neutrophils expressed CD54, there were distinct CD54-high (CD54^hi^) and CD54-low (CD54^lo^) expressing cells, with the majority being CD54^lo^ cells during PICS (Figure 1B). In Unt spleens, however, the CD54-expressing cells were markedly less in number with no distinct separation or difference in the number of CD54^lo^ cells compared to the CD54^hi^ cells (Figure 1B). The percent population comprising CD54^lo^ cells mostly formed a distinct peak from the CD54^hi^ population in PICS spleen unlike the Unt cells as shown in the representative FACS image (Figure 1C). Interestingly, when we compared the neutrophil populations of CD54^hi^ vs. CD54^lo^ in PICS spleens from post-CLP day 2–8, we found that the CD54^hi^ population was significantly higher in the acute phase after infection, but gradually over time, the CD54^lo^ population became the dominant phenotype. By the time all mice develop PICS, the ratio was reversed, and the CD54^lo^ neutrophils were significantly higher than the CD54^hi^ population, unlike in the Unt mice (Figure 1D). The total WBC counts ranged from 86 to 174 million in the control Unt mice and 87 to a much increased number of 552 million in CLP mice starting from day 2 through day 8 post-CLP. Together, these results indicate the appearance of two distinct neutrophil populations in the PICS spleen.

### CD54^hi^ Neutrophils Show Proinflammatory Properties While CD54^lo^ Neutrophils Show Chemotactic and Homing Properties

**Figure 2:**
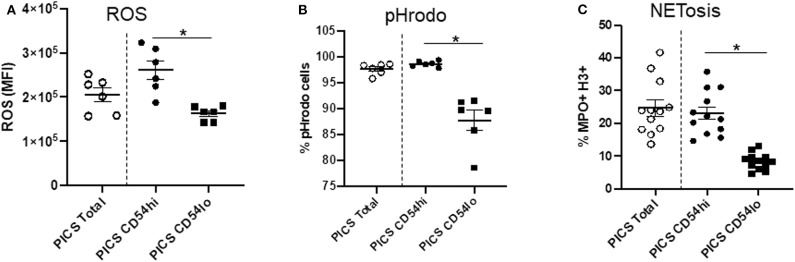
CD54^hi^ neutrophils demonstrate proinflammatory properties. Spleen cells from persistent inflammation, immunosuppression, and catabolism syndrome (PICS) mice were labeled with Ly6G, CD11b, and CD54 and functional assays such as dihydrorhodamine (DHR) to measure reactive oxygen species (ROS), pHrodo (phagocytosis), and NETosis were separately performed using flow cytometry. Scatter plots showing **(A)** ROS mean fluorescence intensity (MFI) of DHR, **(B)** percent pHrodo+ cells, and **(C)** percent MPO+H3+ cells (NETosis) in total, and CD54^hi^ or CD54^lo^ expressing splenic neutrophils. Experiments were repeated at least three times. Black bars in scatter plots indicate the mean ± SEM values. *p < 0.05 was considered significant.

In order to explore the function of the CD54^hi^ and CD54^lo^ cells specifically during PICS, we then evaluated their ability to produce ROS, undergo phagocytosis, and form neutrophil extracellular traps (NETs). A DHR assay was performed to assess ROS production by measuring the oxidation of DHR. CD54^lo^ cells showed significantly decreased ROS production compared to the CD54^hi^ cells as depicted by the mean fluorescence intensity (MFI) in Figure 2A. Moreover, CD54^lo^ cells had significantly decreased phagocytosis and NETosis, compared to CD54^hi^ neutrophils (Figures 2B, **C**).

**Figure 3:**
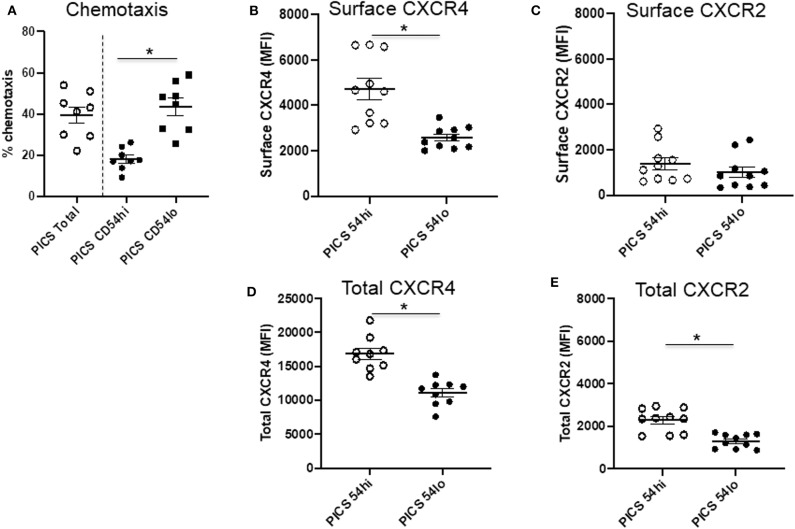
CD54^lo^ neutrophils demonstrate increased chemotactic properties. Spleen cells isolated from persistent inflammation, immunosuppression, and catabolism syndrome (PICS) mice were subjected to either chemotaxis assays and then collected for labeling with Ly6G, CD11b, CD54, or were directly colabeled with CXCR4 and CXCR2 along with Ly6G, CD11b, and CD54 followed by flow cytometry analysis. **(A)** Percent chemotaxis of neutrophils from the spleen of PICS mice were plotted to quantify the chemotaxis in total, as well as in CD54^hi^, and CD54^lo^ cells. Scatter plots showing **(B)** surface expression (MFI) of CXCR4 and **(C)** CXCR2, and **(D)** total expression (MFI) of CXCR4 and **(E)** CXCR2 on CD54^hi^ and CD54^lo^ splenic neutrophils from PICS mice. Experiments were repeated at least twice. Black bars in scatter plots indicate the mean ± SEM values. *p < 0.05 was considered significant.

While these studies indicate that CD54^lo^ cells may be less proinflammatory in nature, this population exhibited greater chemotactic ability compared to CD54^hi^ cells (Figure 3A). To evaluate this further, the expressions of surface and total CXCR4 and CXCR2 were examined in both populations. The majority of CXCR4 expression on neutrophils in the spleen during PICS was intracellular, as evidenced by significantly more total CXCR4 relative to surface CXCR4 (mean total MFI value, 13,854 ± 4,849 vs. mean surface MFI, 3,034 ± 684). However, CD54^lo^ neutrophils had greatly reduced surface as well as total CXCR4 expression, compared with CD54^hi^ cells (Figures 3B,D). For CXCR2, the majority of expression was on the surface, as evidenced by almost similar total and surface levels of CXCR2 (mean total MFI value, 1,537 ± 444 vs. mean surface MFI, 1,321 ± 826). While both CD54^hi^ and lo cells had equivalent surface expression of CXCR2, the total levels were decreased in CD54^lo^ cells, indicating a lower availability of CXCR2 receptors to recycle back to the surface after stimulation (Figures 3C, **E**). Altogether, these results indicated a higher chemotactic and homing ability but less inflammatory function of CD54^lo^ cells, whereas CD54^hi^ cells have greater inflammatory function with decreased chemotactic responses during PICS.

### CD54^hi^ Cells Are the First Proliferative Population While CD54^lo^ Cells Engage in Late Cycling

**Figure 4:**
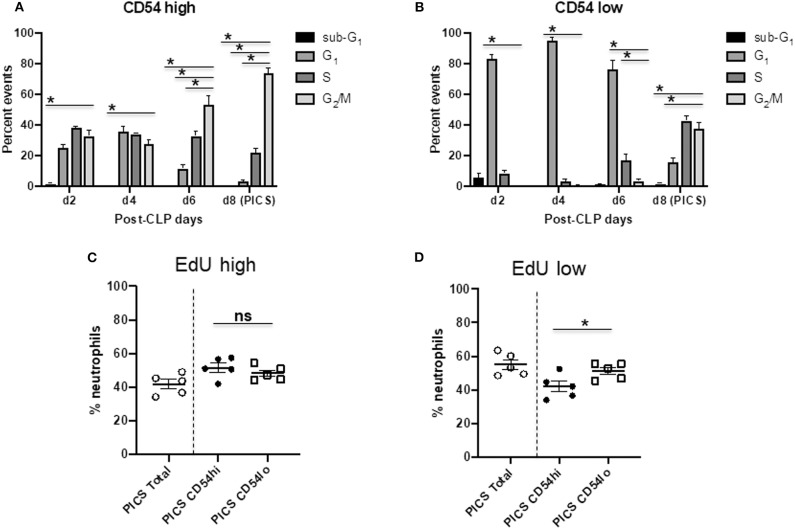
CD54^hi^ cells are the first proliferative population while CD54^lo^ cells engage in late cycling. Spleen cells from mice at days 2, 4, 6, and 8 (d2, d4, d6, and d8, respectively) after CLP were colabeled with Ki-67 and PI along with Ly6G, CD11b, and CD54 to quantify the cell cycle phases by Ki-67 vs. PI gating using flow cytometry. Bar diagrams depicting percent events of sub-G_1_ G_1_, S, and G_2_/M phases in **(A)** CD54^hi^ and **(B)** CD54^lo^ neutrophils from post-CLP mice. Each bar represents the mean ± SEM of percent events of each cell cycle phase at respective post-CLP day from mouse spleen after CLP (*n* = 5–8). 5-Ethynyl-2′-deoxyuridine (EdU) assays on neutrophils from the spleens of persistent inflammation, immunosuppression, and catabolism syndrome (PICS) mice were then performed as described earlier. Scatter plots showing **(C)** percent neutrophils with high EdU incorporation (EdU high) after completion of S phase, and **(D)** low EdU incorporation (EdU low) with an ongoing S phase in total vs. CD54^hi^ and CD54^lo^ splenic cells in PICS mice. Experiments were repeated at least twice. Black bars in scatter plots indicate the mean ± SEM values. *p < 0.05 was considered significant. ns, non-significant.

Next, we investigated the proliferation and cell cycle distribution of the CD54 high and low populations to assess whether they could differentially contribute to emergency myelopoiesis. To examine the cell cycle status of proliferating neutrophils, we analyzed the cells for the proliferation-specific marker, Ki-67, as well as for DNA content by propidium iodide (PI) staining using flow cytometry (22). The Ki-67^+^ population included the active cell cycle phases (G_1_, S, and G_2_/M), while the quiescent or resting (G_0_) cells were negative in Ki-67. PI vs. Ki-67 gating was used to identify the distribution of CD54^hi^ and CD54^lo^ cells in sub-G_1_ (apoptotic cells with fragmented DNA), G_1_, S, G_2_/M, and G_0_ phases from mouse spleens post-CLP days 2–8 (Figure S2). No G_0_ event was detected in either of the cell populations, indicating that all neutrophils had entered the active cell cycle phases following infection (Figure S2). CD54^hi^ cells were found cycling until post-CLP day 6, when the majority of the cells were found in G_2_/M, with some in S phase, but the least in G_1_ phase. By day 8, both S and G_1_ events were further decreased, the lowest being in G_1_, while the maximum (>80%) were in G_2_/M (Figure 4A). This suggests that all the cycling cells gradually reached G_2_/M with no further recycling or entry of new cells into G_1_ by day 8 after CLP. On the other hand, CD54^lo^ neutrophils showed an almost opposite pattern of cell cycle kinetics from post-CLP days 2–8. The majority of events (>80%) was paused in the G_1_ phase during post-CLP days 2–4 until around post-CLP day 6, when the CD54^lo^ population started progressing from G_1_ to S phase (Figure 4B). The transition of CD54^lo^ cells further continued through G_1_-S–G_2_/M phases post-CLP day 8 (Figure 4B).

We also used the EdU incorporation method to detect and quantify the proliferating cells in CD54^hi^ and CD54^lo^ populations during PICS. Mice were injected with EdU on day 7 after CLP, and cells were harvested for analysis after 24 h on post-CLP day 8. Both populations had cells that finished maximum incorporation of EdU (EdU high) after a full S phase (Figure 4C), CD54^hi^ being slightly higher (~4%) than CD54^lo^ cells but not statistically significant. Interestingly, cells that did not finish the S phase yet (ongoing S) and incorporated comparatively lesser EdU (EdU low) by post-CLP day 8 were significantly higher (~10%) in CD54^lo^ compared to the CD54^hi^ population (Figure 4D). This result also supported our previous cell cycle data showing that the CD54^hi^ population gradually completed the S phase and progressed to the next phase (G_2_/M) of the cycle, while the CD54^lo^ population started actively cycling post-CLP days 6–8 (Figures 4A, **B**). Taken together, these data suggest that immediately after CLP, CD54^hi^ cells may be participating in emergency myelopoiesis, as they were proliferating more in the acute phase of infection (post-CLP days 2–4). On the other hand, CD54^lo^ cells, which started cycling at day 6 post-CLP, may be involved in extramedullary myelopoiesis at later time points (post-CLP days 6–8). This again supported our finding that CD54^lo^ cells comprised the majority of neutrophils by day 8 post-CLP (Figure 1D) compared to the CD54^hi^ cells that gradually decreased over time.

### CD54^lo^ Neutrophils Are Not Suppressor Cells in PICS

**Figure 5:**
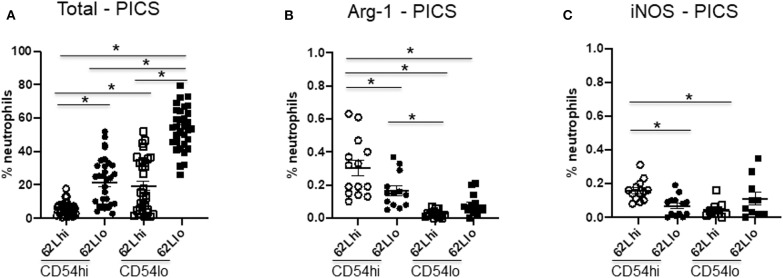
CD54^lo^ neutrophils are not suppressor cells. Neutrophils with high or low CD54 expression (CD54^hi^ and CD54^lo^) from the spleen of persistent inflammation, immunosuppression, and catabolism syndrome (PICS) mice were further characterized based on the adhesion marker, CD62L, and the intracellular expression levels of arginase 1 (Arg-1) and inducible nitric oxide synthase (iNOS) as markers for myeloid-derived suppressor cells using flow cytometry analysis. Scatter plots showing **(A)** percent neutrophils of each subtype, **(B)** percent neutrophils with arginase-1, and **(C)** iNOS expression in CD62L vs. CD54 subpopulations in PICS. Experiments were repeated three times. Black bars in scatter plots indicate the mean ± SEM values. *p < 0.05 was considered significant.

As indicated above, CD62L expression may help determine the level of maturity of neutrophils. In addition, it has been reported that a subset of CD62L^dim^ neutrophils can serve as myeloid-derived suppressor cells (MDSCs) of granulocytic origin and can lead to immunosuppression via a Mac-1 (CD11b/CD18) or ROS-dependent manner (23). When we evaluated the CD54^hi^ and CD54^lo^ neutrophil subsets based on their CD62L expression in PICS mice, we found that CD54^lo^CD62L^lo^ subset was significantly highest among all other subsets (Figure 5A). On the other hand, CD54^hi^CD62L^lo^ neutrophils were significantly less and possibly comprised the minor population of CD54^hi^-activated neutrophils that already shed the ectodomain of CD62L. In concordance, this population also showed the greatest CD11b expression (data not shown), indicating that these are more mature neutrophils. However, the other CD62L^lo^CD54^lo^ cells showed significantly less CD11b expression compared to the CD54^hi^CD62L^lo^ cells. As expected, all CD62L^hi^ subsets showed comparatively less CD11b expression than the CD62L^lo^ cells, again signifying that CD62L can help identify the maturation phase of neutrophils.

As some studies have indicated, emergency myelopoiesis may lead to the excessive release of MDSCs from the bone marrow, which may contribute to the immunosuppression seen in later phases after sepsis (24, 25). Therefore, we further examined other MDSC markers, such as intracellular arginase-1 (Arg-1) and iNOS (26, 27). However, our data indicated that < 1% of neutrophils in the spleen of PICS mice express Arg-1 or iNOS (Figures 5B,C). While both subsets of CD54^lo^ neutrophils had significantly decreased Arg-1 and iNOS expression, the total numbers of each of these cell types are negligible and likely not clinically significant (Figures 5B,C). Taken together, our results indicated that the comparatively immature CD54^lo^ neutrophils are not MDSCs but do have decreased overall immune functions.

## Discussion

This study intended to better characterize the immune function of the splenic neutrophil populations temporally after CLP to enhance the knowledge and understanding of the pathogenesis of PICS. Using our PICS murine model, we found two discrete neutrophil populations in the spleen. One population being the mature CD54^hi^ cells with traditional proinflammatory features that decreased significantly after CLP, and the other being CD54^lo^ cells that were less mature, had decreased inflammatory properties and dominated during the PICS phase. CD54^lo^ neutrophils were also more chemotactic and were actively proliferating, whereas the CD54^hi^ cells stopped reentering the cell cycle for further proliferation during PICS. None of these neutrophils showed any suppressor activity but were less functional with reduced inflammatory responsiveness. Our current study has identified a unique extramedullary CD54^lo^ neutrophil population in spleen characterized by reduced immune function during PICS that may explain the pathophysiology in sepsis-induced chronic critical illness.

Neutrophil heterogeneity can be phenotypic or functional and is pronounced at different levels of their life cycle, either in homeostatic or disease conditions (13, 28). Infectious inflammation can induce rapid changes in neutrophil variants as a function of maturity or activation state (11, 28). While the innate immune response is the initial responders to infection, the other cellular response are also important. It turns out that neutrophils, being the primary defenders of innate response, also interact with other cell types (particularly T cells) and can really shape the ensuing responses, both acutely and over time. In addition, it is known that the myeloid-derived suppressor cells (MDSCs), which are typically considered immature, may also comprise the neutrophil population (11, 29). In our PICS murine model, the early proliferation of CD54^hi^ cells immediately after acute infection suggested an emergency myelopoiesis, while the late onset of cell cycle in the gradually dominating CD54^lo^ phenotype in the spleen suggested an ongoing extramedullary myelopoiesis. However, the contribution of these differential neutrophil populations to the immunosuppression seen in the later stages of sepsis is not known. Earlier studies have indicated that the immunosuppression in septic patients might result from the expansion of persistent MDSCs immediately after the emergency myelopoiesis, which may result in chronic critical illness [reviewed in (30)]. However, in our study, we found a newly emerging population in PICS spleen—the population of CD54^lo^ neutrophils with decreased immune function. It has been shown that, in CLP mice 7 days post-sepsis, up to 95% BM cells are myeloid cells mostly immature and function like MDSCs, which gradually evolve with time to become more immunosuppressive and infiltrate the spleen, lymph nodes, lung, liver, skeletal muscle, and brain (2). MDSCs are generally granulocytic (CD11b+ Ly6G+) and monocytic (CD11b+ Ly6G– Ly6C+) cells. We chose to evaluate the granulocytes in spleen to gain a better sense of the changes in immune cells peripherally, than just simply measuring peripheral blood neutrophils and monocytes, which would not necessarily describe the happenings within remote tissues systemically. MDSCs have been so far mostly implicated in immunosuppression in sepsis while they can also be proinflammatory potentially damaging to parenchymal cells. Interestingly, in our study, we found that the proinflammatory granulocytic cells (CD11b+ Ly6G+ CD54^hi^) were immunoresponsive but more mature and less in number, while the dominant population comprised the newly proliferating chemotactic granulocytes (CD11b+ Ly6G+ CD54^lo^) lacking immune responsiveness. These CD54^lo^ cells also included the major subpopulation CD54^lo^CD62L^lo^, which had comparatively lower CD11b expression than the functional CD54^hi^CD62L^lo^ cells and did not express intracellular Arg-1 or iNOS, indicating that these cells were not part of the MDSC community. Previously, other reports suggested a distinct human neutrophil phenotype in the blood during acute inflammation, characterized by CD54^bright^ cells (CD62L^dim^/CD16^bright^/CD11b^bright^/CD54^bright^) showing immune suppression capacity via T cell suppression (23). The effect of CD54^hi^ and CD54^lo^ cells on T cell function in our study, however, is not yet known.

Neutrophils capable of migration from the bone marrow after granulopoiesis or of reverse transmigration to the bone marrow for further homing are known to have down-regulated CXCR4 expression through decreased CXCR4/CXCL12 signaling (5). While decreased CXCR2 is associated with neutrophil adhesion (31), neutrophils lacking both CXCR4 and CXCR2 are known to display constitutive mobilization, with CXCR4 playing the dominating role in neutrophil trafficking (32). In our study, the CD54 populations showed overall decreased CXCR2 expression. It is well-known that the recruitment of mature and immature neutrophils from the bone marrow occurs to establish a niche in the spleen (33). However, the CD54^lo^ cells significantly lacked CXCR4 expression, which may indicate a greater transmigration or homing ability of this population.

This current study is only limited to a mouse model of PICS. Therefore, it is necessary to expand this study to patients with chronic critical illness. In addition, we do not yet know the role of these differential populations of splenic neutrophils in the setting of a secondary infection. Therefore, although the CD54^lo^ cells have decreased inflammatory functions, further studies are required to determine if they contribute to the immunosuppression that occurs in PICS. Ongoing studies in our lab will reveal more information about the status and function of circulating neutrophils in both the early and later stages of sepsis and help determine the exact role of splenic neutrophils in the development of chronic critical illness after sepsis. Furthermore, focused studies will be interesting to investigate the role of any similar or other population of neutrophils and/or other innate immune cells in tissues other than spleen in PICS.

In conclusion, the comparatively immature, actively proliferating neutrophils arising in spleen have significantly less proinflammatory function, yet preserved chemotactic ability during PICS, which may act as a contributing factor of immunosuppression as seen after sepsis. Therapeutic strategies to target these neutrophils might benefit critically ill sepsis survivors and improve overall outcomes for this patient population.

## Data Availability Statement

All datasets generated for this study are included in the article/**Supplementary Material**.

## Ethics Statement

This animal study was reviewed and approved by Institutional Animal Care and Use Committee of the University of Cincinnati (Protocol # 10-05-10-01).

## Author Contributions

SS: design of the work, data collection, data analysis and interpretation, drafting the article, critical revision of the article, and final approval of the version to be published. CC and VN: conception of the work, data analysis and interpretation, drafting the article, critical revision of the article, and final approval of the version to be published.

## Conflict of Interest

The authors declare that the research was conducted in the absence of any commercial or financial relationships that could be construed as a potential conflict of interest.
