# Supplementary figures and images for: Distinct Neutrophil Populations in the Spleen During PICS
Source: Front Immunol. 2020 May 15;11:804. doi: 10.3389/fimmu.2020.00804 (PMC7243340; doi:10.3389/fimmu.2020.00804)

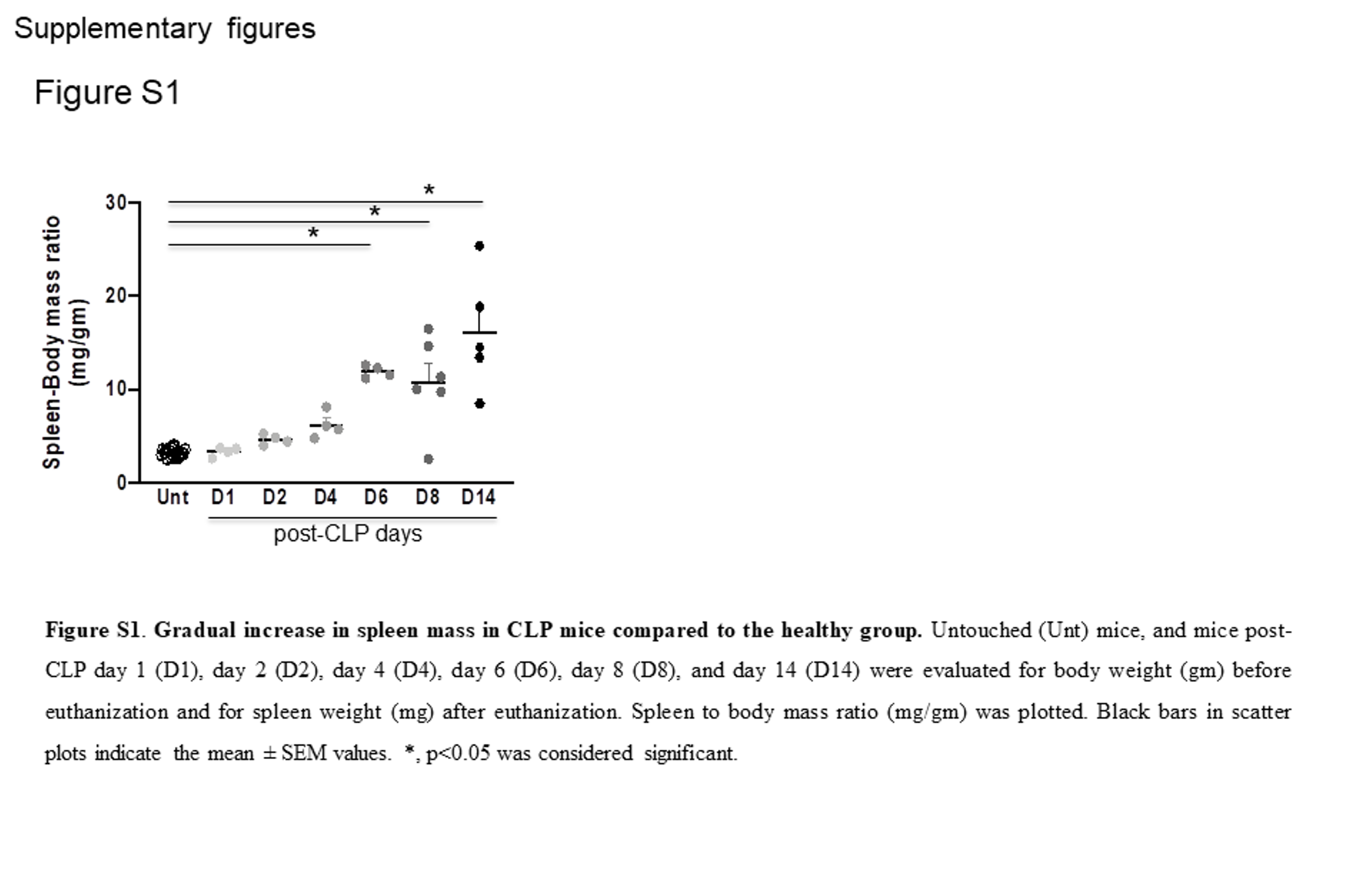

Supplement: Supplementary file 1 [file Image_1.TIF]

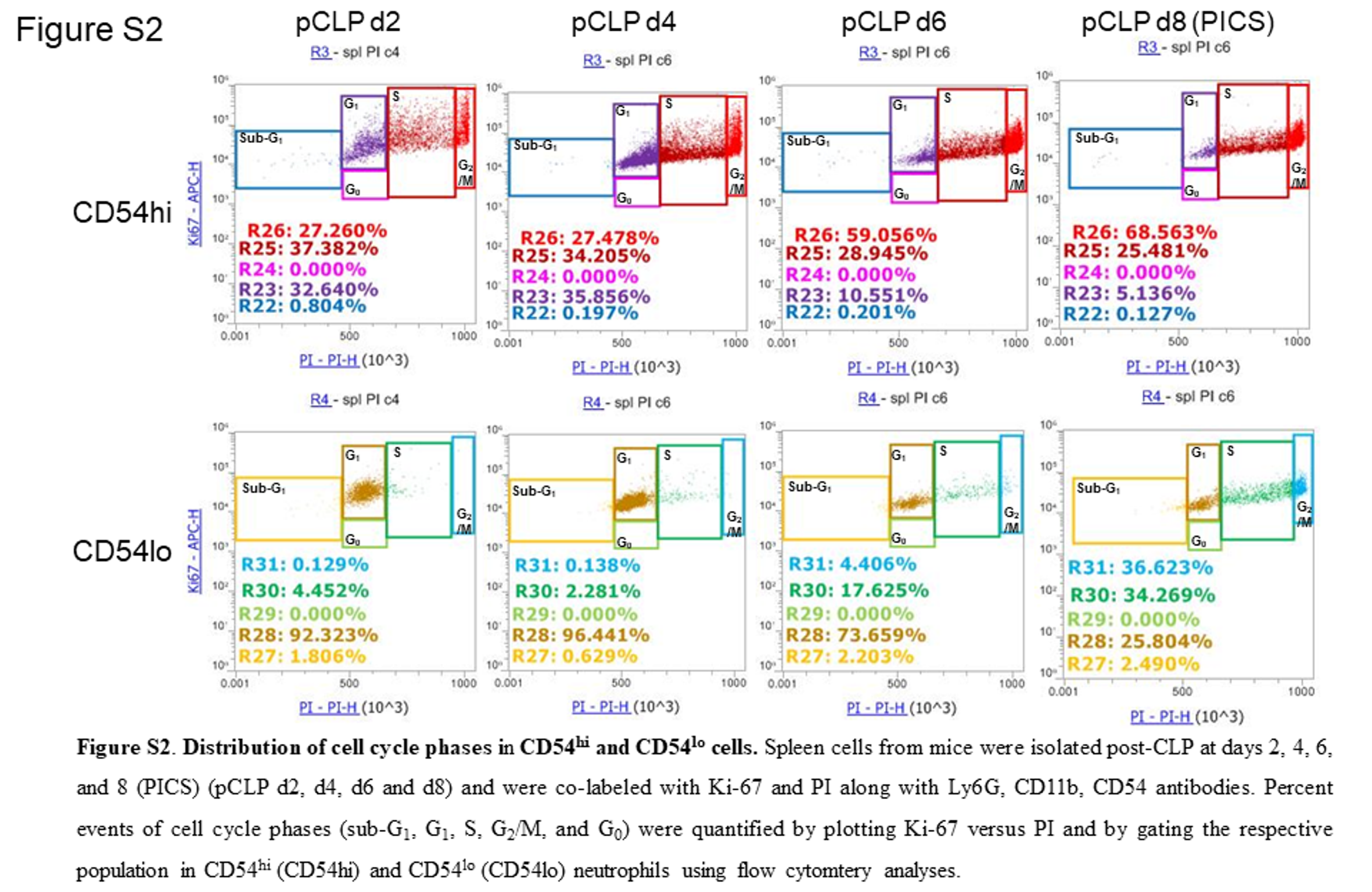

Supplement: Supplementary file 2 [file Image_2.TIF]
